# Supplementary material for: Multi-label annotation of text reports from computed tomography of the chest, abdomen, and pelvis using deep learning
Source: BMC Med Inform Decis Mak. 2022 Apr 15;22:102. doi: 10.1186/s12911-022-01843-4 (PMC9011942; doi:10.1186/s12911-022-01843-4)
Supplement: Supplementary file 1 — Additional file 1: Appendix S1. Dictionary terms used in this study. [file 12911_2022_1843_MOESM1_ESM.docx]

| **Appendix 1**. Dictionary terms used in this study. | | | |
| --- | --- | --- | --- |
|  | **Lungs/Pleura** | **Liver/Gallbladder** | **Kidneys/Ureters** |
| **Organ Anatomy** | lung, pulmonary, lower\|upper\|middle lobe, centrilobular, perifissural, left\|right base, bases, basilar, bronch, trachea, airspace, airway | liver, hepatic, hepato, gallbladder, thegallbladder, gall bladder, biliary, bile, left\|right\|caudate\|quadrate lobe | kidney, renal, nephr, ureter, cort, medul, caliectasis, UVJ |
| **Single-organ**  **Disease descriptors** | pneumothorax, emphysema, pneumoni, ground glass, aspiration, bronchiectasis, atelecta, embol, air trapping, pleural effusion, pneumonectomy | steatosis, cirrho, cholecystectomy, gallstone, cholelithiasis | hydronephrosis, hydroureter, nephrectomy, pelvicaliectasis, uropathy, ureterectasis, nephrolithiasis |
| **Multi-organ Disease Descriptors** | mass, opaci, calcul, stone, scar, metas, malignan, cancer, tumor, neoplasm, lithiasis, atroph, recurren, hyperenhanc, hypoenhanc, aneurysm, lesion, nodule, nodular, calcifi, opacit, effusion, resect, thromb, infect, infarct, inflam, fluid, consolidate, degenerative, dissect, collaps, fissure, edema, cyst, focus, angioma, spiculated, architectural distortion, lytic, pathologic, defect, hernia, biops, encasement, fibroid, hemorrhage, multilocul, distension, stricture, obstructi, hypodens, hyperdens, hypoattenuat, hyperattenuat, necrosis, irregular, ectasia, destructi, dilat, granuloma, enlarged, abscess, stent, fatty infiltr, stenosis, delay, carcinoma, adenoma, atrophy, hemangioma, density, surgically absent | | |
| **Negation** | no, non, other, not, none, without, rather, negative, with regards to, however is no, are no, no evidence, noevidence, limited exam for the evaluation | | |
| **Qualifiers** | acute, new, size, contour, attenuation, caliber, however, morphological | | |
| **Normal** | Normal, unremarkable, negative exam, patent, clear, no abnormalit, without abnormalit | | |
